# 5-Eth­oxy-1,3,4-thia­diazole-2(3*H*)-thione

**DOI:** 10.1107/S1600536812002024

**Published:** 2012-01-21

**Authors:** Sung Kwon Kang, Nam Sook Cho, Siyoung Jang

**Affiliations:** a Department of Chemistry, Chungnam National University, Daejeon 305-764, Republic of Korea

## Abstract

In the title compound, C_4_H_6_N_2_OS_2_, the dihedral angle between the five-membered heterocyclic ring and the plane of the eth­oxy group is 4.9 (2)°. The 1,3,4-thiadiazole-2-thione unit is planar, with an r.m.s. deviation of 0.011 Å from the corresponding squares plane defined by the seven constituent atoms. In the crystal, pairs of N—H⋯S hydrogen bonds link the mol­ecules into inversion dimers.

## Related literature

For the synthesis and reactivity of thia­diazole derivatives, see: Hildebrandt *et al.* (2011 ▶); Zhan *et al.* (2009 ▶); Cho *et al.* (1998 ▶); Squillacote & Felippis (1994 ▶); Antolini *et al.* (1993 ▶).
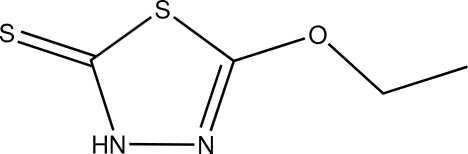


## Experimental

### 

#### Crystal data

C_4_H_6_N_2_OS_2_
*M* *_r_* = 162.23
Triclinic, 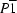

*a* = 6.0308 (12) Å
*b* = 8.1171 (16) Å
*c* = 8.7616 (18) Å
α = 116.55 (4)°
β = 93.70 (3)°
γ = 106.10 (3)°
*V* = 359.7 (2) Å^3^
*Z* = 2
Mo *K*α radiation
μ = 0.66 mm^−1^
*T* = 296 K
0.16 × 0.12 × 0.08 mm

#### Data collection

Bruker SMART CCD area-detector diffractometer
Absorption correction: multi-scan (*SADABS*; Bruker, 2002 ▶) *T*_min_ = 0.905, *T*_max_ = 0.951
10973 measured reflections
1329 independent reflections
1020 reflections with *I* > 2σ(*I*)
*R*_int_ = 0.078

#### Refinement

*R*[*F* ^2^ > 2σ(*F* ^2^)] = 0.035
*wR*(*F* ^2^) = 0.106
*S* = 1.03
1329 reflections
86 parameters
H atoms treated by a mixture of independent and constrained refinement
Δρ_max_ = 0.34 e Å^−3^
Δρ_min_ = −0.25 e Å^−3^

### 

Data collection: *SMART* (Bruker, 2002 ▶); cell refinement: *SAINT* (Bruker, 2002 ▶); data reduction: *SAINT*; program(s) used to solve structure: *SHELXS97* (Sheldrick, 2008 ▶); program(s) used to refine structure: *SHELXL97* (Sheldrick, 2008 ▶); molecular graphics: *ORTEP-3* (Farrugia, 1997 ▶); software used to prepare material for publication: *WinGX* (Farrugia, 1999 ▶).

## Supplementary Material

Crystal structure: contains datablock(s) global, I. DOI: 10.1107/S1600536812002024/is5053sup1.cif

Structure factors: contains datablock(s) I. DOI: 10.1107/S1600536812002024/is5053Isup2.hkl

Supplementary material file. DOI: 10.1107/S1600536812002024/is5053Isup3.cml

Additional supplementary materials:  crystallographic information; 3D view; checkCIF report

## Figures and Tables

**Table 1:** Hydrogen-bond geometry (Å, °)

*D*—H⋯*A*	*D*—H	H⋯*A*	*D*⋯*A*	*D*—H⋯*A*
N3—H3⋯S6^i^	0.76 (2)	2.57 (2)	3.317 (3)	170 (3)

Symmetry code: (i) 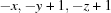.

**Fig. 1.**
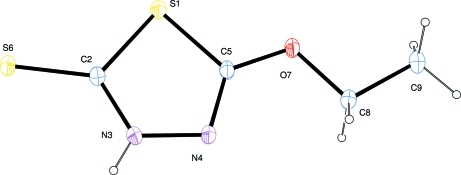
Molecular structure of the title compound, showing the atom-numbering scheme and 30% probability ellipsoids.

**Fig. 2.**
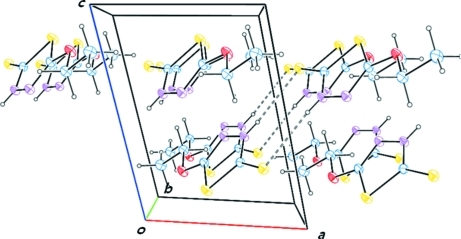
Part of the crystal structure of the title compound, showing molecules linked by intermolecular N—H···S hydrogen bonds (dashed lines).

## supplementary crystallographic information

### Comment

Thiadiazole derivatives have recently attracted attention in synthesis and biological activities (Hildebrandt *et al.*, 2011; Zhan *et al.*, 2009). 1,2,4-Thiadiazolidine-3,5-dione is a 5-membered analog of uracil on the basis of the well known subject between a –CH=CH– group in benzenoid hydrocarbons and the divalent sulfur in its sulfur containing counterparts. 5-Thioxo-1,3,4-thiadiazolidin-2-one is an analog of 1,2,4-thiadiazolidine-3,5-dione (Squillacote & Felippis, 1994; Antolini *et al.*, 1993). Derivatives of 5-thioxo-1,3,4-thiadiazolidin-2-one have potential to have biological activities. The title compound, 5-ethoxy-3*H*-1,3,4-thiadiazoline-2-thione (I) is an intermediate to prepare 3-thioxo-1,3,4-thiadiazolidin-2-one through hydrolysis. However, the hydrolysis afforded bis(2-oxo-3*H*-1,3,4-thiadiazolinyl)-5,5'-disulfide which is a oxidative dimer of 5-thioxo-1,3,4-thiadiazolidin-2-one (Cho *et al.*, 1998)

The 1,3,4-thiadiazole-2-thione unit is planar, with an r.m.s. deviation of 0.011 Å from the corresponding squares plane defined by the seven constituent atoms. The bond distance of N4—C5 [1.293 (3) Å] is shorter than that of C2—N3 [1.325 (3) Å], which is consistent with double bond character. The intermolecular N3—H3···S6^i^ [symmetry code: (i) -*x*, -*y* + 1, -*z* + 1] hydrogen bonds link two molecules into a centrosymmetric dimer (Fig. 2 and Table 1), which stabilize the crystal structure.

### Experimental

Ethyl thiocarbazinate (11.6 g, 0.1 mol) was dissolved in CS_2_ (6.5 ml, 0.11 mol). KOH (0.86 g, 18 mmol) in 20 ml of methyl alcohol was added to the above solution and it was refluxed for 6 h. The reaction mixture was cooled to room temperature and distilled off the solvent under reduced pressure. The resulting residue was dispersed in 20 ml water and acidified with c-HCl (9 ml). Product was collected (8.0 g, 51% yield) and recrystallized in benzene to obtain the analytical sample. Colourless crystals of (I) were obtained from its ethanol solution by slow evaporation of the solvent at room temperature. mp 128–130 °C, Rf, 0.48 (hexane: ethyl acetate = 7: 3 *v*/*v*); IR (KBr, cm^-1^) 3100 (NH), 2850 (CH), 1560 (C=N), 1350. ^1^H NMR (CDCl_3_, p.p.m.) 13.7 (1*H*, b, NH), 4.4 (2*H*, q, CH_2_), 1.4 (3*H*, t, CH_3_).; ^13^C NMR (CDCl_3_, p.p.m.) 184.2 (C=S), 165.6 (C—O), 69.0 (CH_2_), 14.0 (CH_3_). Anal. Calcd. for C_4_H_6_N_2_OS_2_: C 29.62, H 3.73, N 17.27. Found: C 29.75, H 3.58, N 16.56.

### Refinement

Atom H3 of the NH group was located in a difference Fourier map and refined freely. Other H atoms were positioned geometrically and refined using a riding model, with C—H = 0.97 or 0.96 Å, and with *U*_iso_(H) = 1.2*U*_eq_(carrier C) for methylene or 1.5*U*_eq_(carrier C) for methyl H atoms.

### Crystal data

**Table:**

C_4_H_6_N_2_OS_2_	*Z* = 2
*M**_r_* = 162.23	*F*(000) = 168
Triclinic, *P*1	*D*_x_ = 1.498 Mg m^−^^3^
Hall symbol: -P 1	Mo *K*α radiation, λ = 0.71073 Å
*a* = 6.0308 (12) Å	Cell parameters from 3632 reflections
*b* = 8.1171 (16) Å	θ = 2.9–24.6°
*c* = 8.7616 (18) Å	µ = 0.66 mm^−^^1^
α = 116.55 (4)°	*T* = 296 K
β = 93.70 (3)°	Block, colourless
γ = 106.10 (3)°	0.16 × 0.12 × 0.08 mm
*V* = 359.7 (2) Å^3^	

### Data collection

**Table:**

Bruker SMART CCD area-detector diffractometer	1020 reflections with *I* > 2σ(*I*)
graphite	*R*_int_ = 0.078
φ and ω scans	θ_max_ = 25.4°, θ_min_ = 2.7°
Absorption correction: multi-scan (*SADABS*; Bruker, 2002)	*h* = −7→7
*T*_min_ = 0.905, *T*_max_ = 0.951	*k* = −9→9
10973 measured reflections	*l* = −10→10
1329 independent reflections	

### Refinement

**Table:**

Refinement on *F*^2^	Primary atom site location: structure-invariant direct methods
Least-squares matrix: full	Secondary atom site location: difference Fourier map
*R*[*F*^2^ > 2σ(*F*^2^)] = 0.035	Hydrogen site location: inferred from neighbouring sites
*wR*(*F*^2^) = 0.106	H atoms treated by a mixture of independent and constrained refinement
*S* = 1.03	*w* = 1/[σ^2^(*F*_o_^2^) + (0.0692*P*)^2^] where *P* = (*F*_o_^2^ + 2*F*_c_^2^)/3
1329 reflections	(Δ/σ)_max_ < 0.001
86 parameters	Δρ_max_ = 0.34 e Å^−^^3^
0 restraints	Δρ_min_ = −0.25 e Å^−^^3^

### Special details

**Table:**

Geometry. All e.s.d.'s (except the e.s.d. in the dihedral angle between two l.s. planes) are estimated using the full covariance matrix. The cell e.s.d.'s are taken into account individually in the estimation of e.s.d.'s in distances, angles and torsion angles; correlations between e.s.d.'s in cell parameters are only used when they are defined by crystal symmetry. An approximate (isotropic) treatment of cell e.s.d.'s is used for estimating e.s.d.'s involving l.s. planes.
Refinement. Refinement of *F*^2^ against ALL reflections. The weighted *R*-factor *wR* and goodness of fit *S* are based on *F*^2^, conventional *R*-factors *R* are based on *F*, with *F* set to zero for negative *F*^2^. The threshold expression of *F*^2^ > σ(*F*^2^) is used only for calculating *R*-factors(gt) *etc*. and is not relevant to the choice of reflections for refinement. *R*-factors based on *F*^2^ are statistically about twice as large as those based on *F*, and *R*- factors based on ALL data will be even larger.

### Fractional atomic coordinates and isotropic or equivalent isotropic displacement parameters (Å^2^)

**Table:**

	*x*	*y*	*z*	*U*_iso_*/*U*_eq_	
S1	0.58072 (11)	0.69466 (8)	0.86851 (8)	0.0633 (3)	
C2	0.3257 (4)	0.6379 (3)	0.7233 (3)	0.0478 (5)	
N3	0.2660 (4)	0.4510 (3)	0.6039 (3)	0.0522 (5)	
H3	0.158 (4)	0.408 (4)	0.531 (3)	0.055 (8)*	
N4	0.3981 (3)	0.3405 (3)	0.6130 (3)	0.0523 (5)	
C5	0.5704 (4)	0.4537 (3)	0.7487 (3)	0.0500 (6)	
S6	0.18884 (11)	0.79469 (9)	0.73697 (8)	0.0591 (3)	
O7	0.7344 (3)	0.4010 (2)	0.8031 (2)	0.0623 (5)	
C8	0.7080 (5)	0.1955 (3)	0.7051 (3)	0.0572 (6)	
H8A	0.7218	0.1607	0.5857	0.069*	
H8B	0.5544	0.1142	0.7027	0.069*	
C9	0.9014 (5)	0.1656 (4)	0.7956 (4)	0.0711 (7)	
H9A	0.89	0.0306	0.7338	0.107*	
H9B	0.8853	0.1999	0.9133	0.107*	
H9C	1.0524	0.2471	0.7974	0.107*	

### Atomic displacement parameters (Å^2^)

**Table:**

	*U*^11^	*U*^22^	*U*^33^	*U*^12^	*U*^13^	*U*^23^
S1	0.0619 (4)	0.0459 (4)	0.0679 (4)	0.0255 (3)	−0.0096 (3)	0.0151 (3)
C2	0.0472 (12)	0.0499 (13)	0.0521 (12)	0.0229 (10)	0.0101 (10)	0.0260 (10)
N3	0.0476 (11)	0.0498 (11)	0.0560 (12)	0.0221 (9)	−0.0011 (10)	0.0215 (9)
N4	0.0525 (11)	0.0445 (10)	0.0592 (11)	0.0241 (9)	0.0025 (9)	0.0216 (9)
C5	0.0480 (13)	0.0453 (12)	0.0593 (14)	0.0223 (10)	0.0042 (11)	0.0249 (11)
S6	0.0597 (4)	0.0531 (4)	0.0668 (4)	0.0319 (3)	0.0059 (3)	0.0248 (3)
O7	0.0583 (10)	0.0458 (9)	0.0739 (11)	0.0241 (8)	−0.0102 (8)	0.0214 (8)
C8	0.0620 (15)	0.0471 (13)	0.0634 (14)	0.0263 (11)	0.0047 (11)	0.0243 (11)
C9	0.0803 (19)	0.0673 (17)	0.0799 (18)	0.0429 (14)	0.0091 (14)	0.0382 (14)

### Geometric parameters (Å, °)

**Table:**

S1—C5	1.738 (2)	O7—C8	1.449 (3)
S1—C2	1.740 (2)	C8—C9	1.502 (3)
C2—N3	1.325 (3)	C8—H8A	0.97
C2—S6	1.665 (2)	C8—H8B	0.97
N3—N4	1.377 (3)	C9—H9A	0.96
N3—H3	0.76 (2)	C9—H9B	0.96
N4—C5	1.293 (3)	C9—H9C	0.96
C5—O7	1.321 (2)		
			
C5—S1—C2	89.00 (11)	O7—C8—C9	107.07 (19)
N3—C2—S6	127.91 (18)	O7—C8—H8A	110.3
N3—C2—S1	107.12 (17)	C9—C8—H8A	110.3
S6—C2—S1	124.97 (15)	O7—C8—H8B	110.3
C2—N3—N4	120.52 (19)	C9—C8—H8B	110.3
C2—N3—H3	118 (2)	H8A—C8—H8B	108.6
N4—N3—H3	121 (2)	C8—C9—H9A	109.5
C5—N4—N3	107.33 (18)	C8—C9—H9B	109.5
N4—C5—O7	125.7 (2)	H9A—C9—H9B	109.5
N4—C5—S1	116.01 (17)	C8—C9—H9C	109.5
O7—C5—S1	118.33 (16)	H9A—C9—H9C	109.5
C5—O7—C8	115.95 (17)	H9B—C9—H9C	109.5

### Hydrogen-bond geometry (Å, °)

**Table:**

*D*—H···*A*	*D*—H	H···*A*	*D*···*A*	*D*—H···*A*
N3—H3···S6^i^	0.76 (2)	2.57 (2)	3.317 (3)	170 (3)

Symmetry codes: (i) −*x*, −*y*+1, −*z*+1.
